# A phase I trial of Capecitabine+Gemcitabine with radical radiation for locally advanced pancreatic cancer

**DOI:** 10.1038/sj.bjc.6604827

**Published:** 2008-12-16

**Authors:** M Michael, T Price, S Y Ngan, V Ganju, A H Strickland, A Muller, K Khamly, A D Milner, J Dilulio, A Matera, J R Zalcberg, T Leong

**Affiliations:** 1 Division of Haematology and Medical Oncology, Peter MacCallum Cancer Centre, Locked Bag 1, A’Beckett Street, Melbourne, Victoria 8006, Australia; 2 Department of Medical Oncology, Queen Elizabeth Hospital, 28 Woodville Road, Woodville South, South Australia 5011, Australia; 3 Department of Medical Oncology, Lyell McEwin Health Service, Haydown Road, Elizabeth Vale, South Australia 5112 Australia; 4 Division of Radiation Oncology, Peter MacCallum Cancer Centre, Locked Bag 1, A’Beckett St, Melbourne, Victoria 8006, Australia; 5 Department of Medical Oncology, Frankston Hospital, Hastings Road, Frankston, Victoria 3199, Australia; 6 Department of Medical Oncology, Monash Medical Centre, Moorabbin Campus Centre Road, East Bentleigh, Victoria 3165, Australia; 7 Department of Radiation Oncology, Flinders Medical Centre, Flinders Drive, Bedford Park, South Australia 5042, Australia; 8 Biostatistics and Clincial Trial Centre, Peter MacCallum Cancer Centre, Locked Bag 1, A’Beckett Street, Melbourne, Victoria 8006, Australia

**Keywords:** Capecitabine, Gemcitabine, pancreatic cancer, chemoradiotherapy

## Abstract

Standard chemoradiotherapy with infusional 5FU for locally advanced pancreatic cancer (LAPC) has limited efficacy in this disease. The combination of Capecitabine (Cap) and Gemcitabine (Gem) are synergistic and are potent radiosensitisers. The aim of this phase I trial was thus to determine the highest administered dose of the Cap plus Gem combination with radical radiotherapy (RT) for LAPC. Patients had LAPC, adequate organ function, ECOG PS 0–1. During RT, Gem was escalated from 20–50 mg m^−2^ day^−1^ (twice per week), and Cap 800–2000 mg m^−2^ day^−1^ (b.i.d, days 1–5 of each week). Radiotherapy 50.4 Gy/28 fractions/5.5 weeks, using 3D-conformal techniques. Three patients were entered to each dose level (DL). Dose-limiting toxicity(s) (DLTs) were based on treatment-related toxicities. Twenty patients were accrued. Dose level (DL) 1: Cap/Gem; 800/20 mg m^−2^ day^−1^ (3 patients), DL2: 1000/20 (12 patients), DL3: 1300/30 (5 patients). Dose-limiting toxicities were observed in DL3; grade 3 dehydration (1 patient) and grade 3 diarrhoea and dehydration (1 patient). Dose level 2 was the recommend phase 2 dose. Disease control rate was 75%: PR=15%, SD=60%. Median overall survival was 11.2 months. The addition of Cap and Gem to radical RT was feasible and active and achieved at relatively low doses.

The treatment of unresectable locally advanced pancreatic cancer (LAPC) is challenging (Verslype *et al*, 2007), given it is characterised by a median survival of 8–12 months (Kim and Saif, 2007). The treatment options have included palliative surgery, chemotherapy or chemoradiotherapy (CRT) (Huguet *et al*, 2007; Verslype *et al*, 2007). The combination of radiotherapy (RT) with infusional 5FU has been considered by many as the standard of care. The pivotal trials have shown a survival advantage of CRT relative to RT or chemotherapy alone (Moertel *et al*, 1981), albeit inconsistently (Cohen *et al*, 2005). A recent meta-analysis has also confirmed the significant survival advantage of CRT here (Sultana *et al*, 2007).

Nevertheless, alternative drugs for radiosensitisation and systemic control are required to improve outcome. Gemcitabine (Gem) is a potent radiosensitiser, (Lawrence *et al*, 1996) and a number of phase I trials have thus far combined this agent with RT for LAPC (Blackstock *et al*, 1999; Talamonti *et al*, 2000; Wolff *et al*, 2001; Epelbaum *et al*, 2002; Yamazaki *et al*, 2004; Pipas *et al*, 2005; Brade *et al*, 2007). A phase III trial in patients with LAPC has also directly compared Gem alone *vs* weekly Gem concurrent with radical radiation therapy followed by further Gem therapy (Loehrer *et al*, 2008).

Studies in pancreatic cell lines (Lawrence *et al*, 1996), and in murine squamous cell carcinoma models (Fields *et al*, 2000), have confirmed that minimal cytotoxic doses are required for radiosensitivity and which persists for 48 h after administration. Hence, drug administration more than once per week during RT may be beneficial (Lawrence *et al*, 1996; Fields *et al*, 2000; Blackstock *et al*, 2003; Brade *et al*, 2007).

The utility of Capecitabine (Cap) with radiation in LAPC has also been assessed. The final step in its activation to 5FU is catalysed by thymidine phosphorylase (TP). Higher levels of TP are found in malignant cells resulting in preferential tumoral activation, and its expression has been correlated with an objective response to Cap-based regimens (Eda *et al*, 1993). Thymidine phosphorylase is also upregulated by cytotoxics, as well as radiation (Sawada *et al*, 1999). It has been combined with RT for LAPC in a number of trials using various schedules (Schneider *et al*, 2005; Crane *et al*, 2006; Saif *et al*, 2007). The combination of Cap and Gem has also shown superiority to Gem alone in a phase III trial of patients with advanced disease (Cunningham *et al*, 2005).

Given the synergy between Gem and Cap and their respective radiosensitisation, the primary objective of this phase I trial was therefore to determine for the first time the highest administered dose of oral Cap combined with twice weekly Gem and radical RT in patients with LAPC. The secondary objectives were to determine its safety profile and response.

## Patients and methods

### Patient selection

#### Inclusion criteria

The following patients were eligible: (i) histological/cytological confirmation of LAPC that was either deemed unresectable by specialist hepatobiliary surgeon(s) due to disease extent or recurrent following surgical resection; (ii) disease suitable for radical RT (primary tumour <5 cm diameter); (iii) measurable or evaluable disease; (iv) age ⩾18 years; (v) ECOG performance status 0–1; (vi) adequate organ function: bone marrow – haemoglobin ⩾90 g l^−1^, neutrophil count ⩾1.5 × 10^9^ l^−1^, platelet count ⩾100 × 10^9^ l^−1^; hepatic – serum bilirubin ⩽1.5 × upper limit normal (ULN), AST and/or ALT ⩽3.0 × ULN; renal – creatinine clearance ⩾50 ml min^−1^; (vii) written informed consent.

#### Exclusion criteria

The following patients were ineligible: (i) distant metastases, (ii) prior chemotherapy or RT, (iii) significant underlying medical condition(s) that may be aggravated by the study treatment, (iv) conditions associated with the inability to swallow, tolerate or absorb oral medication.

The trial protocol was approved by the Ethics Committees of the treating institutions.

### Radiation therapy

#### Total dose and technique

A total of 50.4 Gy in 28 fractions, 1.8 Gy/fraction, 5 fractions a week, in 5.5 weeks was planned. Patients were treated using 3D-conformal techniques in accordance with ICRU50/62 recommendations. All fields were treated on treatment days with 6 MeV or higher energy machine.

#### Radiotherapy target volumes

The gross tumour volume (GTV) was defined on axial CT slices and included the gross primary tumour and involved lymph nodes (>1 cm). During the period of this study, routine IV or oral contrast was not given during the radiotherapy-planning scan. However, the GTV was delineated with the aid of diagnostic CT scans performed with IV and oral contrast.

The clinical target volume (CTV)=GTV. Prophylactic irradiation of uninvolved lymph node regions was not undertaken.

The planning target volume (PTV)=CTV+1 cm.

#### Dose constraints to critical organs at risk

The following dose constraints were used: (i) kidney; dose to one-third of either kidney was not to exceed 35 Gy, dose to two-thirds of either kidney not to exceed 20 Gy, and the mean dose to either kidney should not to exceed 23 Gy; (ii) liver; dose to one-third of liver was not to exceed 50 Gy, dose to two-thirds of liver not to exceed 35 Gy, and the mean dose to liver not to exceed 31 Gy; (iii) spinal cord; the maximum dose to the spinal cord was not to exceed 45 Gy.

#### Simulation

Patients were simulated in the supine position. Simulation of CT with CT slices at 5 mm intervals from top of T11 vertebra to the bottom of L4. Dose volume histograms for liver and kidney were generated.

#### Verification

Beam eye view simulation films were required for angled fields. Weekly check films were performed.

#### Dose modifications for radiotherapy

Radiotherapy, together with chemotherapy, was suspended for any grades 3–4 radiation-associated toxicities, and recommenced once they had improved to ⩽grade 1. Further chemotherapy, at reduced dose, was not given unless considered to be in the patient's best interest. If the RT was interrupted for 2 or more weeks, all therapy was ceased.

### Chemotherapy

#### Concurrent chemotherapy and dose escalation

Cap was given orally twice a day from Monday to Friday. The morning dose was taken approximately 2 h before RT, with the second dose 12 h afterwards. Doses omitted because of toxicity were not replaced.

Gem was to be given twice a week, on Monday and Thursday of RT, administered over 30 min within 2 h of delivery of RT. Antinauseant premedication was given as per institutional practice.

Both Cap and Gem were planned to be dose escalated in up to 8 dose levels (DLs), with doses ranging from 800 to 2000 mg m^−2^ day^−1^ (b.i.d, Monday to Friday) and 20–50 mg m^−2^ (Monday and Thursday), respectively. Chemotherapy after RT was not mandated.

#### Dose-limiting toxicities

Dose-limiting toxicities were defined prospectively using the NCI-CTC (version 2, April 1999), and based on toxicities experienced during and within 2 weeks following CRT. The DLTs were: (i) grade 4 neutropenia (ANC <0.5 × 10^9^ l^−1^) of any duration, (ii) grade 4 thrombocytopenia (platelet count <10 × 10^9^ l^−1^) or grade 3 thrombocytopenia (platelet count 10–49 × 10^9^ l^−1^) with bleeding, (iii) febrile neutropenia, (iv) grade 3 or 4 non-haematological toxicity (within or outside the radiation field) including nausea and vomiting despite adequate therapy, but excluding alopecia, (v) toxicity requiring ⩾1 dose reductions during CRT, (vi) >1 week delay in RT.

#### Dose escalation

Three patients were planned to be entered into each DL and the dose escalation/expansion schema was standard. If no DLTs were observed in these three patients, the next DL was opened. If DLTs were observed in one of three patients, then three additional patients (total of six) were accrued at this DL. If DLTs were observed in one of six patients, the next DL was opened. If DLTs were observed in ⩾2 of 3 or 6 patients, no further dose escalation occurred.

Escalation to the next DL occurred once all three or six patients had reached the 2-week point after CRT. The highest administered dose was defined as that DL in which two or more of three or six patients had DLTs. The recommended phase 2 dose (RP2D) level (1 below the highest administered dose) was expanded to a total of 12 patients to obtain further data regarding toxicities and response.

#### Dose modifications for concurrent chemotherapy

During CRT, the chemotherapy doses were modified based on the worst toxicity grades. Treatment modification was in two forms, either permanent dose reductions or treatment deferral with the recommencement at a reduced dose on recovery. Patients entered at DL1 requiring more than 1 dose reduction or entered at higher DLs requiring more than 2 dose reductions discontinued chemotherapy, but the RT continued.

Treatment was stopped early due to either disease progression, a >2 week delay in RT delivery, unacceptable toxicity or patient request.

### Monitoring procedures and tests

Within 4 weeks of trial entry, patients underwent a CT scan of the chest/abdomen/pelvis, with dedicated pancreatic views. Patients were not staged by endoscopic ultrasound. Within 1 week of trial entry, patients had blood taken for haematology (full blood examination (FBE)), biochemistry (creatinine, urea and electrolytes, liver function tests) and Ca19-9.

During CRT, patients were evaluated weekly for toxicity by both radiation and medical oncologists, blood taken for biochemistry (weekly) and FBE (Mondays and Thursdays). Compliance with study medications was also assessed. On completion of treatment, patients were reviewed two weekly for 6 weeks for toxicities, and restaged with CT scan, and Ca19-9 level. Patients were subsequently reviewed every 2 months until progression.

### Statistical analysis

Patient baseline characteristics, treatment delivery details and protocol deviations were summarised using descriptive statistics. Acute toxicities graded using the NCI-CTC (version 2.0, 30 April 1999) and late radiation toxicities graded using the RTOG criteria were summarised by DL. Response was assessed by the RECIST criteria. The best response to treatment, sites of relapse or progression and status at last follow-up were summarised for each DL.

Overall survival (OS) was measured from the date of protocol treatment commencement to the date of death from any cause. The Kaplan–Meier product limit method was used to estimate OS, with censoring of survival times at the close-out (censor) date for those patients still alive. Statistical analyses were performed using S-Plus 2000 Professional software (MathSoft Inc., Seattle, WA, USA, 1999).

## Results

### Patients

A total of 20 patients were recruited from between November 2002–August 2006, across five centres. Their demographics are summarised in Table 1. The cohort was recruited across DL1-3, as follows: (a) DL1 (Cap 800 mg m^−2^day^−1^, Gem 20 mg m^−2^), three patients, (b) DL2 (Cap 1000 mg m^−2^ day^−1^, Gem 20 mg m^−2^), initially three patients and then expanded to a total of 12 patients and (c) DL3 (Cap 1300 mg m^−2^ day^−1^, Gem 30 mg m^−2^), five patients.

### Treatment delivery

#### Radiotherapy

Fourteen patients (70%) received protocol treatment; 3 of 3 from DL1, 7 of 12 from DL2 and 4 of 5 from DL3. One patient (DL2) received an additional two fractions of RT to a total dose of 54 Gy/30 fractions. Five patients (25%) received fewer than the protocol specified 28 fractions of RT, the reasons were as follows:

- DL2: (i) one patient, 19.8 Gy/11 fractions, due to opiate-induced collapse and pneumonia resulting in admission and omission of all treatment from week 3; (ii) one patient, 37.8 Gy/21 fractions, 4 day break in RT due to nausea (grade 3), diarrhoea (grade 2) and pain; (iii) one patient each missed 1 and 2 fractions, respectively, for unspecified reasons.
- DL3: one patient, 36.0 Gy/20 fractions due to lethargy and diarrhoea (grade 3).

#### Concurrent chemotherapy

Dose level 3 was identified as the highest administered DL. Hence, DL2 was identified as the RP2D and expanded to a total of 12 patients.

Nine patients (45%) received all planned chemotherapy; 2 of 3 from DL1, 6 of 12 from DL2 and 1 of 5 from DL3. The dose omission/reductions were as follows:

- DL1: one dose of Gem omitted on day 1, week 6.
- DL2: (i) one patient: Cap ceased from week 5 due to grade 3 nausea and grade 2 diarrhoea; (ii) one patient: Cap omitted in weeks 4 and 5 due to grade 2 nausea; (iii) two patients for other non-trial-related reasons; (iv) one patient for nausea and vomiting in week 1 and diarrhoea in weeks 2 and 4; (v) one patient with one dose of Gem omitted (week 2) by own choice.
- DL3: (i) one patient due to non-neutropenic grade 2 fever in week 5 of CRT, chemotherapy was discontinued but RT was completed; (ii) one patient due to grade 3 diarrhoea in week 1, with a dose reduction of Cap in weeks 2 and 3 and then its cessation; (iii) one patient with one dose of Cap omitted in weeks 5 and 6, and one dose of Gem omitted in week 4 (public holiday) and in week 6; (iv) one patient with one dose each of Gem and Cap omitted in week 2 due to public holiday.

The average overall relative dose intensity (actual dose delivered as a percentage of the planned dose) was 90.8% (s.d.=20.6%) for Cap and 92.3% (s.d.=18.0%) for Gem. At the RP2D, the relative dose intensity was 92.6% (s.d.=20.1%) for Cap and 94.7% (s.d.=19.9%) for Gem.

### Dose-limiting toxicities

Dose-limiting toxicities were reported in two patients, both in DL3: (i) one patient with treatment-related grade 3 dehydration, onset 38 days from the start of treatment and (ii) one patient with grade 3 diarrhoea, onset 4 days from the start of treatment, with a dose reduction of Cap in weeks 2 and 3 and its cessation subsequently. Dose level 3 was thus identified as the highest administered dose.

### Toxicity

The acute haematological and non-haematological toxicities from day 1 of CRT till 6 weeks post-completion are detailed in Tables 2 and 3, respectively and were not unexpected. Overall, the CRT was well tolerated, where two patients (10%) had suffered grade 3 diarrhoea and one patient (5%) each nausea, vomiting and abdominal pain.

Late radiation toxicities were reported in two patients: one patient in DL1 with the onset of grade 1 small/large intestine toxicity 148 days from the start of the RT, and the other patient in DL2 with onset of grade 2 liver toxicity 121 days from the start of RT. The latter patient had acute hepatic toxicity, with an elevated ALP and AST (12.4 × and 2.5 × ULN, respectively) 3 weeks post-completion of treatment. These changes persisted till 12 weeks post-treatment (6.3 × and 1.4 × ULN, respectively) and eventually normalised by 16 weeks. There were no related symptoms.

### Response

Three patients (15%) had a partial response (one at DL1 and two at DL3), 12 patients (60%) had a response of stable disease and four (20%) had progressive disease; one patient was non-evaluable (Table 4).

Overall, 15 patients were evaluable for Ca-19-9 response. Two patients (13.3%) had achieved ⩾50% reduction relative to the baseline value, 10 patients (67.7%) had stabilisation (<50% reduction to <25% increase) and 3 patients (20%) had progression (>30% increase).

### Survival parameters

**Figure 1:**
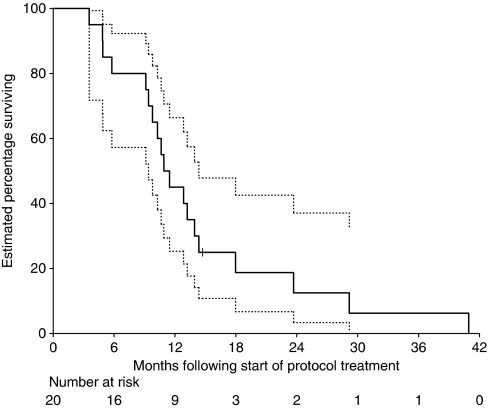
Kaplan–Meier curve of overall survival for all 20 patients. Ninety-five percent confidence intervals are shown by dotted lines. Patients with censored times are shown by tick marks.

One patient of the 20 was still alive on the close-out date of 29 October 2007. The median OS was estimated at 11.2 months (95% confidence interval 9.4–14.4 months) (Figure 1).

### Sites of relapse

Ten patients (50%) were reported to have relapsed or progressed, five involving distant metastases, three involving primary relapse, one involving nodal relapse and one combined primary and distant failure. Five patients received further chemotherapy on progression.

## Discusssion

Given the synergy of Gem and Cap combinations (Cunningham *et al*, 2005), and their respective radiosensitisation, the aim of this trial was to identify for the first time the highest dose of these agents given concurrently with radical radiation in patients with LAPC. In this trial, DL2 (Cap 1000 mg m^−2^ day^−1^, Gem 20 mg m^−2^ 2 × per week) was determined to be the recommended DL, achieving a relative dose intensity of over 90% for both agents with good overall tolerance. It must be noted that the doses achieved here in this combination are approximately 50–60% of that achieved when each agent has been given alone with radical RT (Blackstock *et al*, 1999, 2003; Saif *et al*, 2005, 2007; Brade *et al*, 2007; Loehrer *et al*, 2008).

It must also be noted that Gem has marked schedule dependency in terms of its toxicity profile and maximal tolerated dose: whether given alone or in combination with radiotherapy (Martin *et al*, 1996). Studies in experimental models (Lawrence *et al*, 1996; Fields *et al*, 2000) have confirmed that minimal cytotoxic doses are required for radiosensitivity and persist for 48 h after administration. Hence, drug administration more than once per week during RT, as given in this trial, may be beneficial (Lawrence *et al*, 1996; Fields *et al*, 2000; Blackstock *et al*, 2003; Brade *et al*, 2007). However other schedules of Gem have been combined with external beam radiotherapy (50.4 Gy) as discussed above. This has included a weekly regimen, where doses ranging from 250–600 mg m^−2^ per week have been evaluated. These doses approach active systemic exposures of the agent, but as well, may potentially provide greater radiosensitisation with 50.4 Gy radiation compared with a twice weekly schedule (Epelbaum *et al*, 2002; Okusaka *et al*, 2004; Cengiz *et al*, 2007; Loehrer *et al*, 2008).

The inability to dose escalate was not a reflection of the trial design. The dose escalation plan here was standard, the DLTs were pragmatic and corresponded to clinically significant treatment-related toxicities and the patients were carefully reviewed. This inability was most likely due to these agents providing at least an additive radiosensitisation when given together with radical radiation.

There are other examples of this in the literature. A recent phase I trial attempted to dose escalate Gefitinib, a potent radiosensitiser, and Cap with RT in patients with LAPC (Czito *et al*, 2006). In this study, three of seven patients at DL1 (Gefitinib 250 mg day^−1^, Cap 650 mg m^−2^/b.i.d. continuously) and all of three at DL2 (250 mg day^−1^ and 825 mg m^−2^/b.i.d.) developed a DLT comprising of grades 3–4 gastrointestinal toxicity (Czito *et al*, 2006). Similarly, a phase I trial combined twice weekly Gem and cisplatin for 3 weeks during RT (50.4 Gy/28 fractions) in patients with LAPC or gastric cancer (Martenson *et al*, 2003). The highest tolerated dose was DL5 (Gem 30 mg m^−2^ and cisplatin 10 mg m^−2^) (Martenson *et al*, 2003). It thus appears that the dose escalation of two active cytotoxic radiosensitisers is limited in the setting of CRT for LAPC, possibly related to the dose-limiting normal structures within the target volume.

The regimen was overall well tolerated and the toxicity profile concurs with CRT studies using either agent alone (Table 3) (Blackstock *et al*, 1999, 2003; Ben-Josef *et al*, 2004; Saif *et al*, 2005; Brade *et al*, 2007). The low rates of GI toxicity observed reflected the weekly review during CRT, the DL achieved and importantly the use of 3D-conformational radiation fields in an effort to spare the dose-limiting normal structures including the small bowel. Although not directly shown in this study, it is more likely that the use of such multifield radiation techniques will produce less toxicity compared with anteroposterior–posteroanterior techniques used in some previously reported studies (Talamonti *et al*, 2000; Wolff *et al*, 2001; Goldstein *et al*, 2007). The treatment of smaller volumes covering only gross disease is also more likely to produce less toxicity compared with larger volumes that include prophylactic irradiation of uninvolved regional lymph nodes.

In a phase I trial by ECOG, seven patients with LAPC were treated with radiation (59.4 Gy) and infusional 5FU with Gem doses escalated from 50 to 100 mg m^−2^ per week. Three patients developed upper gastrointestinal ulceration with severe bleeding, and three of the five episodes of DLT developed at radiation doses ⩽36 Gy (Talamonti *et al*, 2000). The treatment volume encompassed the tumour with a 2-cm margin, and a dose of 45 Gy was delivered to all of the at-risk regional lymph nodes (Talamonti *et al*, 2000). In the trial reported here, a specific 3D-conformal RT technique was used and the RT target volumes did not include the treatment of uninvolved regional lymph nodes.

As this was a phase I trial with a small cohort size, response and survival were not primary end points and hence caution must be given in their interpretation. The response rate was consistent with those reported by CRT trials evaluating bi-weekly Gem alone (Pipas *et al*, 2001; Brade *et al*, 2007; Loehrer *et al*, 2008), or similar Cap regimens (Saif *et al*, 2005), and similarly for survival times (Blackstock *et al*, 1999; Epelbaum *et al*, 2002; Poggi *et al*, 2002; Brade *et al*, 2007; Saif *et al*, 2007; Loehrer *et al*, 2008). Hence, it cannot be concluded that the CRT combination evaluated here provided a greater activity than either agent alone due to limited dose escalation. Further advances may be achieved by the addition of biological agents to enhance radiosensitisation (Krempien *et al*, 2007; Small *et al*, 2008).

Despite achieving local control, the majority of LAPC patients relapse distantly, as observed in this trial. In this cohort, patients were not mandated to have a screening laparascopy to exclude peritoneal disease. It also implies that adequate systemic control and the identification of patients who are more likely to progress early are essential steps to improve survival with this multimodality approach.

Given the limited modest success with current systemic regimens (Cunningham *et al*, 2005; Kindler *et al*, 2007; Moore *et al*, 2007), alternative approaches are required. The identification of patients more likely to respond to chemotherapy or CRT is one approach. At present, there is a paucity of reliable consistent genetic data in this regard (Ogawa *et al*, 2006). In one promising study of patients with resectable pancreatic adenocarcinoma, treated with neoadjuvant concurrent Gem and RT, polymorphisms in the DNA damage response/repair genes were observed to have had a significant impact on the OS (*P*<0.02) (Li *et al*, 2006).

One alternative approach has been the selecting out those patients who would progress early with an upfront CRT approach. For example, initial induction chemotherapy followed by CRT in those patients who had not progressed. A retrospective analysis of patients with LAPC enrolled into two studies compared the survival of those treated with initial chemotherapy and then in the absence of progression who received CRT, with that of patients who continued with chemotherapy alone. After initial control by chemotherapy, CRT had significantly improved survival compared with further chemotherapy alone: the median progression-free survival times were 10.8 *vs* 7.4 months (*P*=0.005), and the median OS times 15.0 and 11.7 months, respectively (*P*=0.0009) (Huguet *et al*, 2007). It is still unclear if induction chemotherapy followed by CRT is superior to CRT alone.

In conclusion, the addition of Cap and Gem to radical RT was feasible and tolerable in patients with LAPC. The recommended dose was Cap 1000 mg m^−2^ day^−1^ and Gem 20 mg m^−2^ day^−1^ (twice per week) when combined with 50.4 Gy. The efficacy of this regimen will require testing in the phase II setting.

## Figures and Tables

**Table 1:** Patient characteristics

**Parameter**	**Category**	***N***	**% (*N*=20)**
Gender	Male	12	60%
	Female	8	40%
Age at registration (years)	Median	64	
	Range	41–80	
Pancreatic subsite	Head	19	95%
	Body	1	5%
T stage^a^	1	2	10%
	2	8	40%
	3	6	30%
	4	4	20%
N stage	0	10	50%
	1a	1	5%
	1b	5	25%
	X	4	20%
Earlier surgery	Yes	17	85%
	No	3	15%
Baseline Ca19-9 (kU l^−1^)	Median	366	
	Range	13–6546	

a UICC TNM 5th edition, 1997.

**Table 2:** Non-haematological toxicities observed during concurrent chemoradiotherapy and 6 weeks post (NCI-CTC Version 2, 30 April 1999)

		**Dose level**			
**Toxicity**	**Worse grade**	**1 (*N*=3)**	**2 (*N*=12)**	**3 (*N*=5)**	**Total (%)**
Diarrhea	3	0	1^a^	1	2 (10)
Fatigue	3	0	1	2	3 (15)
Anorexia	3	0	0	1	1 (5)
Dehydration	3	0	0	2	2 (10)
Nausea	3	0	1	0	1 (5)
Vomiting	3	0	1	0	1 (5)
Abdominal pain	3	0	1	0	1 (5)
Radiation dermatitis	1	0	1	1	2 (10)
Dyspepsia/heartburn	2	1	0	1	2 (10)
Bilirubin	4	0	1	0	1 (5)
ALT	3	0	1	0	1 (5)
Hypokalaemia	3	0	1	3	4 (20)

Note patients may have had more than one concurrent toxicity.

a Not considered as treatment related by the investigator.

**Table 3:** Haematological toxicities observed during concurrent chemoradiotherapy and 6 weeks post (NCI-CTC Version 2, 30 April 1999)

		**Dose level**			
**Toxicity**	**Worse grade**	**1 (*N*=3)**	**2 (*N*=12)**	**3 (*N*=5)**	**Total (%)**
Haemoglobin	2	0	4	2	6 (30)
Platelet count	2	0	2	2	4 (20)
Leucocytes	3	0	0	3	3 (15)
Neutrophils	3	0	0	1	1 (5)

**Table 4:** Radiological response following the completion of all therapies

	**Dose level**			
**Response parameters**	**1 (*N*=3)**	**2 (*N*=12)**	**3 (*N*=5)**	**Total patients (%)**
*Overall best response*				
Complete response	0	0	0	0 (0)
Partial response	1	0	2	3 (15)
Stable disease	2	8	2	12 (60)
Progressive disease	0	3	1	4 (20)
Not evaluable	0	1	0	1 (5)
